# Durable Structural Recycled Concrete for Different Exposure Environments

**DOI:** 10.3390/ma18030587

**Published:** 2025-01-28

**Authors:** Carla Vintimilla, Miren Etxeberria

**Affiliations:** Department of Civil and Environmental Engineering, Campus Nord, Universitat Politècnica de Catalunya·Barcelona TECH, 08034 Barcelona, Spain; carla.vintimilla@upc.edu

**Keywords:** natural and accelerated carbonation, chloride diffusion, chloride permeability, cement types (CEM II/AL, CEM II/AS, CEM III/B), coarse and fine aggregates, supplementary cementitious materials

## Abstract

In this work, the influence of limited percentages of coarse (CRCA) and fine (FRCA) recycled concrete aggregates (Type A recycled aggregates) on the durability properties of structural concrete was analyzed. Concretes were designed using 50% and 60% CRCA with simultaneous additions of 0%, 10%, and 20% FRCA and different types of cement (CEM II/AL 42.5 R, CEM II/AS 42.5 N/SRC, and CEM III/B 42.5 N-LH/SR). Recycled aggregate concrete (RAC) and natural aggregate concrete (NAC) mixtures were produced with similar compressive strength using effective water–cement ratios of 0.47 and 0.5. The drying shrinkage values and durability properties were determined, and they included the chloride permeability, chloride penetration depth, and accelerated and natural carbonation rates. The findings revealed that RAC produced using CEM III/B, which included the mixture produced with 60% coarse RCA and 20% fine RCA, achieved low chloride ion penetrability (up to 850 Coulombs) and exhibited the lowest chloride diffusion coefficient, approximately 7 × 10^−13^. Additionally, the RAC-C60-F20 concretes made with CEM II/AS proved suitable for the XC3 and XC4 exposure environments, guaranteeing a lifespan of 50 and 100 years based on the natural carbonation rate. In addition, the RAC-C60-F20 concrete made with CEM II/AL cement exhibited an adequate natural carbonation rate for XC4 environments, which was between 1.6 and 2.4 units higher than the accelerated carbonation rate. This work validates the use of RAC in XC environments (corrosion induced by carbonation) and XS1 environments (corrosion caused by chlorides from seawater).

## 1. Introduction

Conventional concrete production methods heavily rely on the extraction of natural aggregates (NAs), contributing to habitat degradation, energy consumption, and emissions associated with transportation [1,2]. However, only 8% of the total CO_2_ emissions of cementitious materials are caused by aggregate extraction and use [1,3]. It is known that all production activities for cementitious materials account for approximately 7–8% of the world’s total CO_2_ emissions. Cement, as the critical binding material, constitutes approximately 13% of concrete’s weight and typically 10–15% of the total volume of a concrete product [1,2], while aggregates make up 70 to 80% of the volume of concrete. Thus, incorporating recycled aggregates into concrete production is a viable proposal as a more sustainable alternative for the achievement of a circular economy. While several countries have recommended using coarse recycled concrete aggregate (CRCA) in structural concrete production, the Spanish Structural Concrete Code (SC-BOE) [4] allows for a maximum of 20% replacement with CRCA in concrete with a strength class of C40/50. However, no such provision has been made for fine recycled concrete aggregate (FRCA) due to its influence on the properties of fresh and hardened concrete [5]. The Eurocode 2 (EC-02) [6] allows for up to 40% replacement of the total volume of aggregates using Type-A (FRCA and CRCA) recycled aggregates. RCA (Type-A recycled concrete aggregate) exhibits higher porosity than natural aggregates (NAs) due to adhered mortar and micro-cracks that form during crushing. Thus, the water absorption capacity increases, affecting the amount of water available for mixing. This can lead to issues related to the loss of the concrete’s workability in its fresh state and a decline in the mechanical and durability properties in the long term [7]. The higher drying shrinkage value of recycled aggregate concrete (RAC) than that of natural aggregate concrete (NAC) is due to the presence of adhered mortar and porous material in RCA [8]. The shrinkage value also depends on various factors, including the compressive strength, environmental conditions, and the properties of cement and additions [9]. Early stage shrinkage is particularly critical as it contributes substantially to the final shrinkage magnitude, elevating the risk of cracking in the later stages of concrete’s lifecycle [10,11]. Domingo-Cabo et al. [12] revealed that RAC produced using 20% CRCA exhibits a similar drying shrinkage value to that of NAC. However, RAC using up to 50% and 100% CRAC achieved 20% and 70%, respectively, higher drying shrinkage than that of NAC. Furthermore, Vintimilla and Etxeberria [13] found that structural concretes produced with up to 60% CRCA achieved similar values to those of NAC. Additionally, they verified that the use of up to 20% FRCA together with 60% CRCA also resulted in adequate values ranging up to −800 μm/m according to the ACI (American Concrete Institute) standard [14]. Simsek et al. [15] found that replacing up to 20% with FRCA results in the maintenance of adequate concrete properties after 90 days, but higher percentages significantly increase drying shrinkage. Recent studies confirmed the feasibility of using FRCA in structural concrete with a minimal impact on overall performance [13,16].

The durability of RAC is influenced by the interaction between two main factors. On the one hand, the improvement in the interfacial transition zone (ITZ) between the RCA particles and the new mortar can contribute to increased durability. On the other hand, the higher porosity of RCA tends to increase the total porosity of the concrete, which may reduce its durability. Whether the effects of an improved ITZ or increased porosity predominate will explain the enhanced or diminished durability performance of RAC compared with NAC [17]. Furthermore, variations in the water-to-cement ratio and the type of cement used significantly impact the durability of RAC. Zhao et al. [18] reviewed key durability indices and testing methods, emphasizing the importance of chloride diffusion and freeze-thaw resistance. Similarly, Bu et al. [19] investigated the effects of recycled fine aggregates on permeability and carbonation resistance, underscoring that adjustments to the water–cement ratio can enhance the durability of RAC. The corrosion of reinforcement is a major and complex pathology affecting reinforced concrete structures [20]. The reinforced concrete used in marine infrastructures such as bridges and piers is vulnerable to chloride-induced corrosion, which shortens its service life [21,22]. Seawater chlorides penetrate the concrete, causing significant damage when levels exceed a critical threshold, which is measured using chloride profiles at various depths [23].

The use of structural RAC in chloride-rich environments remains a point of debate due to its higher porosity, which can increase chloride diffusion [24,25,26]. However, reducing the adhered mortar of RCA or using RCA sourced from high-strength concrete can improve resistance to chloride ion penetration [27,28]. Several studies [29,30,31,32,33,34] have demonstrated that the chloride ion diffusion coefficient increases with the proportion of RCA used. In addition, the use of FRCA influences the properties of RAC more than the use of CRCA does. Reducing the water-to-binder ratio or incorporating supplementary cementitious materials (SCMs) such as fly ash or blast furnace slag (BFS) enhances chloride resistance in concrete due to the reduction in the pore size of the paste and the development of an improved ITZ [17,29]. Kirthika and Singh et al. [35] observed that using up to 30% FRCA demonstrated an improvement in resistance to chloride penetration compared with NAC. Li et al. [24] showed that concrete with up to 50% CRCA exhibits low chloride penetrability. However, a higher CRCA content requires the addition of SCMs to ensure sufficient resistance. The addition of BFS further improves chloride resistance through chemical binding via the formation of Friedel’s salt, and chlorides can also accumulate in the diffuse layer of C-A-S-H. This accumulation is usually referred to as physical binding [36,37,38]. Researchers [17,39] have indicated that the influence of RCA on chloride penetration resistance is significantly lower than that of factors such as the w/ratio and the use of SCMs.

Carbonation in concrete is a critical phenomenon that significantly affects the durability and lifespan of concrete structures. The resistance of concrete to carbonation depends on its capacity to bind CO_2_, as well as its porosity and pore size distribution, which are factors that affect its permeability [40]. Natural carbonation occurs as CO_2_ from the atmosphere slowly penetrates the concrete, reacting with calcium hydroxide to form calcium carbonate. This process reduces the pH of the concrete, potentially leading to the corrosion of embedded steel reinforcements over time [40,41]. According to the literature [20,31,42,43], the carbonation depth of RAC increases with the level of natural aggregate replacement. This phenomenon is influenced by several factors, including the increased porosity of RAC due to the higher water absorption (WA) of RCA compared with that of NA, as well as the moisture content and the concentration of CO_2_ in the environment [41]. Several researchers have determined that concrete produced with 50% CRCA exhibits a carbonation rate comparable to that of NAC [44,45,46]. Zeng [44] specifically reported that substituting up to 50% of natural gravel with CRCA did not compromise the carbonation resistance. Evangelista and Brito et al. [31] noted that concrete produced with the total replacement of fine NA with FRCA showed low carbonation resistance. However, the use of up to 30% FRCA and the use of SCM (fly ash) with a higher percentage of FRCA resulted in adequate properties for structural concrete. In addition, despite RCA’s higher porosity, the adhered mortar provides additional reactive content against CO_2_, which can enhance carbonation resistance [47]. However, Pedro et al. [48] determined that when the quality of mortar adhered to RCA was lower than that of a new cement paste, the carbonation rate increased as higher proportions of RCA were used to replace natural aggregates.

Furthermore, it was also demonstrated [20,49,50,51] that RAC using up to 50% CRCA, with a reduction in the effective water–cement ratio without increasing cement amount, could achieve the same strength and carbonation depths as NAC, using superplasticizers to preserve workability. Moreover, with similar compressive strength to that of NAC, RAC showed lower carbonation than NAC when 20–50% replacement was applied [52].

In order to evaluate the carbonation rate of concrete, besides considering its water-to-cement ratio and compressive strength, the employed cement type and additives, such as fly ash or slag, must be considered [40,53,54,55].

The use of SCMs can increase the carbonation rate due to a reduction in total CaO, which decreases the available carbonate components and increases vulnerability to carbonation [34,56]. A study by Greve-Dierfeld et al. [40] demonstrated a greater carbonation depth in mixtures with SCM due to the lower portlandite content. They reduced the Ca availability during cement hydration [35,40], thus decreasing carbonation resistance, altering the material’s porous structure and impacting its diffusivity and permeability. Additionally, as more SCM is used in place of clinker, the service life of concrete decreases [46,55,57]. For example, SCM-containing binders produce less portlandite and contain higher aluminate phases than Portland cement (PC) binders, while retaining unreacted phases allows deeper carbonation ingress compared with PC binders under similar exposure conditions [58].

This study advances the understanding of the durability of RAC produced with type A RCA, using up to 60% CRCA and up to 20% FRCA. The previous work of Vintimilla and Etxeberria et al. [13] established those percentages as viable limits for structural concrete applications while considering the mechanical and deformability properties. Thus, this serves as a point of departure for further exploration of the durability of RAC concretes. The principal objective was to evaluate whether RAC (incorporating 50% and 60% CRCA and 0%, 10%, and 20% FRC; the RCA percentages were verified in previous work [13]) could maintain durability properties comparable to those of NAC when all concretes had similar compressive strengths (RACs and NAC were produced with effective water–cement ratios of 0.47 and 0.51, respectively). In addition, all of the RAC mixtures were produced using three different cement types—CEM II/AL 42.5 R, CEM II/AS 42.5 N/SRC, and CEM III/B 42.5 N-LH/SR.

The properties of the hardened concrete were evaluated experimentally, and they included the compressive strength, drying shrinkage, chloride permeability, chloride profile, and both accelerated and natural carbonation coefficients. The results obtained were compared with values established by specific standards for exposure to XC1–XC4 and XS1 environments to evaluate the feasibility of RAC in structural concrete applications and compare its behavior with that of NAC.

## 2. Materials and Methods

### 2.1. Materials

#### 2.1.1. Cement and Chemical Admixtures

Table 1 illustrates the composition details of three types of cement employed, which are accessible in Barcelona: CEM II/AL 42.5 R, CEM II/AS 42.5 N/SRC, and CEM III/B 42.5 N-LH/SR, with the same minimum compressive strength of 42.5 MPa at 28 days, as defined by the European standard EN 197-1 [59].

In the concrete production process, two chemical admixtures were employed: (1) a superplasticizer (S) based on polycarboxylate ether (PAE) polymer technology and (2) a multifunctional admixture (P) based on modified lignin sulfonate. The dosages of these admixtures recommended by the manufacturer varied between 0.3% to 2% for S and 0.5% to 1.5% for P, based on the weight of cement.

#### 2.1.2. Natural Aggregates

**Figure 1:**
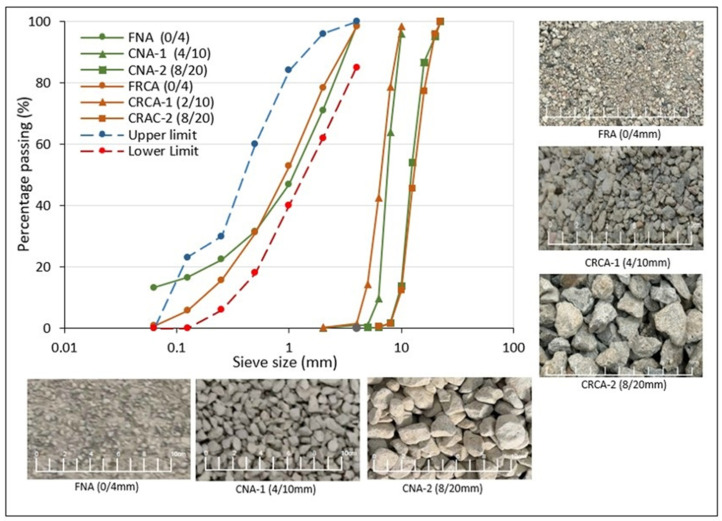
Particle size distribution and geometrical characteristics of all-natural and recycled aggregates.

Natural limestone aggregates, including one fine fraction (FNA, 0/4 mm) and two coarse fractions (CNA-1, 4/10 mm, and CNA-2, 8/20 mm), were employed in concrete production. The geometrical characteristics of these fractions and their grading distribution, determined according to the EN 933-1 [60] and EN12620 [61] specifications, are illustrated in Figure 1. The Structural Concrete Code (SC-BOE) [4] also outlines recommended upper and lower limits for fine aggregate. The dry density and water absorption, which were evaluated according to EN 1097-6 [62], are summarized in Table 2.

#### 2.1.3. Recycled Aggregates

Concrete mixtures were created by utilizing one fine fraction (0/4 mm, FRCA) and two coarse fractions (2/10 mm, CRCA-1, and 8/20 mm, CRCA-2). The production process for the RCA involved crushing, water-cleaning, and sieving C&DW at a recycling plant in Barcelona, Spain [67]. Figure 1 illustrates the shapes and size distributions of the three RCA fractions.

**Figure 2:**
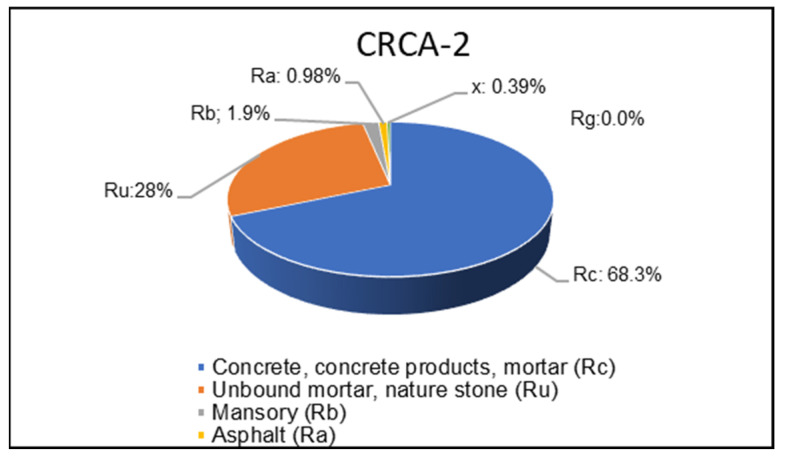
Constituents of type A CRCA-2 (8/20 mm) aggregates.

The composition of the CRCA-2 (8/20 mm) fraction was analyzed according to the EN 933-11 [68] specification and the results are detailed in Figure 2. According to the EN 206 [69] specification, the RAs utilized in this study were categorized as type A (RC90, RCU95, Rb10-, Ra1, FL2-, and XRg1-). Concrete (RC) and natural stone (Ru) components accounted for over 90% of the total content, while the ceramic content was less than 10%. Specifically, the aggregates were classified as RCU95 [13].

Table 2 indicates that the dry density of the RCA fractions exceeded the minimum requirement (2.1 kg/dm^3^) set by EN 206 [69] for concrete use. While the WA of the RCA was higher than that of the NA, it stayed below SC-BOE’s 7% limit. Studies show that RCA’s WA ranges from 3.9–9.6% [70,71] for coarse fractions and 2.4–19.3% [72,73,74] for fine fractions. As previously evidenced in the scientific literature [13,75,76,77], the high WA of RCA is related to the presence of old mortar. The RCA met the standards for the Los Angeles coefficient, sand equivalent, and flakiness index (see Table 2). Alkali-aggregate reactivity analysis of FRCA at 0/4 mm showed less than 0.1% expansion after 14 days, confirming its non-reactivity.

### 2.2. Concrete Production and Test Procedures

#### 2.2.1. Concrete Production

Concrete requires a minimum characteristic design strength (fck) of 30 MPa (C30/37), using a total water–cement ratio of 0.50 and 300 kg of cement, to be able to be used in both XC1–XC4 environments (corrosion induced by carbonation due to humidity) and XS1 environments (corrosion caused by chlorides from seawater) [4]. The effective water–cement ratio of 0.47 was maintained constant in all concrete samples (see Table 3). This effective ratio was determined in conventional concrete (NAC-0.47 concrete) by adjusting the effective amount of water absorbed by aggregates from the total water–cement ratio of 0.50. In addition, a new NAC mixture with an effective w/c ratio of 0.51 was formulated using a total water-to-cement ratio of 0.55 to achieve equivalent compressive strength between RAC and NAC. Although this mix does not meet the minimum total water-to-cement ratio of 0.50 required for XS1 environments, it is valid for XC3–XC4 environments.

The effective amount of water absorbed by aggregates was defined as the amount absorbed by the aggregates within 30 min [13]. The natural fine and coarse aggregates absorbed 70% and 20%, respectively, compared with the 100% and 70% absorbed by FRCA and CRCA, with respect to the absorption capacity measured over 24 h. In order to reduce the absorption capacity of the RCA, it was used in highly humid conditions, with 70–90% of the absorption capacity. The total water content in the concrete was determined by combining effective water and the water within the aggregates (humidity plus the amount of effectively absorbed water).

Table 3 shows the mix proportions of the produced concretes; combinations of 50% and 60% CRCA with 0%, 10% and 20% FRCA were used. The described mixtures were produced using three types of cement (CEM II/AL, CEM II/AS and CEM III/B). As mentioned above, these substitution levels were validated in a previous study by Vintimilla and Etxeberria et al. [13] for structural concrete use.

Table 3 shows the slump values (determined according to the EN 12350-2 [78] specification) of the produced concretes. The concretes made with CEM II/AL exhibited a liquid consistency, and a greater amount of superplasticizer (P) was used than in the corresponding concrete produced with IIAS and IIIB cement. In order to control the consistency, the concretes produced with IIAS and IIIB were produced using a lower superplasticizer content and a slightly higher plasticizer content (1% S and 1% P), achieving a fluid consistency, as defined by the Structural Concrete Code (SC-BOE) [4]. The concrete produced with type IIIB cement achieved a lower slump value than the corresponding concrete produced using IIAS, with the same amount of admixtures. In addition, the concrete produced using FRCA achieved a similar or higher slump value than that of the concrete produced using only CRCA.

The concrete mixtures were prepared in a vertical axis mixer, and the aggregates were added first in order of size. They were mixed for 1 min, after which cement, followed by water and chemical admixtures, was added while the mixing process continued. After mixing for an additional minute, the concrete specimens were manually compacted and covered with plastic for 24-h of curing. Subsequently, the specimens were demolded and stored at 20 ± 2 °C with a relative humidity of ≥95% until 1 h before testing. All of the test elements were kept in the same conditions.

#### 2.2.2. Test Procedure

Table 4 describes the test performed in this study.

**Table 4:** Standards and test procedures for the evaluation of concrete properties.

Parameter	Method	Samples	Information
Compressive Strength	EN 12390-3	3 cubic specimens measuring 100 × 100 × 100 mm, at 7, 28, and 56 days	Determination of the concrete’s load-bearing capacity using a compressive machine (capacity 3000 kN). The machine was configured to accommodate specimens and the load was applied uniformly at a rate of 0.5 MPa/s until failure.
Drying Shrinkage	EN 12390-16	2 specimens measuring 75 × 75 × 280 mm, monitored for 91 days	Determination of the concrete’s length and weight variations over time under controlled conditions. Environment: Controlled climate room at 20 ± 2 °C and 50 ± 5% RH. The specimens were demoulded at 24 h post-casting, weighed, and had their mass recorded, and the reference length (L0) was measured and documented. Subsequently, the specimens were placed in the drying room or chamber for the duration of the test.
Chloride Permeability	ASTM C1202	2 concrete disks (100Ø mm diameter, 50 mm thickness) at 28 and 56 days	Assessment of concrete’s resistance to chloride ion penetration. A potential difference of 60 V was applied across each side of the specimen, which was immersed in solutions containing sodium hydroxide (NaOH) and sodium chloride (NaCl). Measurement of the total charge (in Coulombs) passed during a 6-h testing period.
Chloride Profile	EN 12390-11	Cubic specimens, 100 × 100 mm (Figure 3)	Determination of the chloride content at different depths across 8 layers (L1: 0–1, L2: 1–3, L3: 3–5, L4: 5–7, L5: 7–10, L6: 10–13, L7: 13–16, L8: 16–20 mm) in the depth of the concrete specimen. After curing, the specimens were exposed to a 3% NaCl solution for 91 days in a controlled environment at 23 ± 2 °C and 50 ± 5% relative humidity to simulate the conditions of Atlantic seawater, as per standard laboratory tests for concrete designed for marine environments [22]. The chloride concentration was measured using titration with a 0.02 N silver nitrate solution.
Accelerated Carbonation Resistance	EN 12390-12	2 prismatic specimens of 100 × 100 × 300 mm, measured at intervals 0, 14, 28, 56, 70 and 91 days	Evaluation of concrete’s ability to withstand the carbonation process. Specimens were cured for 28 days in a humidity chamber, followed by 14-days of pre-conditioning at 20 ± 2 °C, 50–55% humidity, and a CO_2_ concentration of 425 ppm. Subsequently, it was exposed to 3% CO_2_ and 57% RH in a 20 °C chamber. The carbonation depth was determined using a solution of 1 g phenolphthalein in 70 g ethanol and 30 g water, as per UNE-EN 14630.
Natural Carbonation Resistance	UNE 83993-1	2 prismatic specimens of 100 × 100 × 400 mm, measured at intervals 0, 30, 90, 180 and 365 days	The specimens were cured for 4 days in a humidity chamber and then placed in a suitable plastic box in an outdoor environment. Solution: 1 g of phenolphthalein in 70 g of ethanol and 30 g of water, as per UNE-EN 14630.

**Figure 3:**
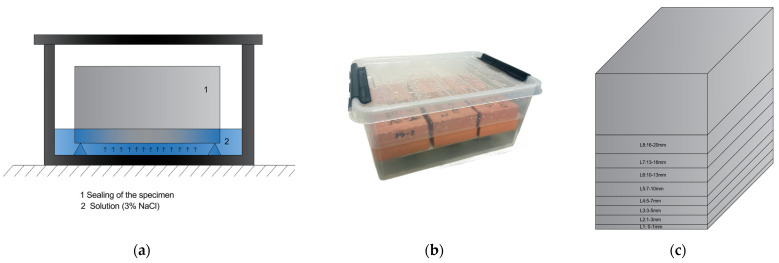
Schematic diagram of chloride profile. (**a**) Schematic of a concrete specimen partially submerged in a solution. (**b**) Photograph of the specimens; (**c**) The depth layer to be analyzed.

## 3. Results and Discussion

### 3.1. Hardened Properties

#### 3.1.1. Compressive Strength

The compressive strength values (fcm, cub_100_) and their standard deviations for cubic concrete specimens (100 × 100 × 100 mm) are presented in Table 5. The specimens, designed for exposure classes XC1–XC4 and XS1 with strength class C30/37, must meet the minimum characteristic and average strength values of 30 MPa and 38 MPa, respectively, for cylindrical specimens (150 × 300 mm), according to the Structural Concrete Code (SC-BOE) [4]. According to the differences in geometries and stress distributions between cubes and cylinders, Vintimilla and Etxeberria et al. [13] determined, in compliance with SC-BOE [4,79], that the minimum average compressive strength for 100 mm cubes (fcm, cub_100_) should be 46 MPa at 28 days.

In addition, the standard deviations were acceptable, with a higher dispersion at 7 days that decreased by 28 and 56 days, indicating reliable measurements.

**Figure 4:**
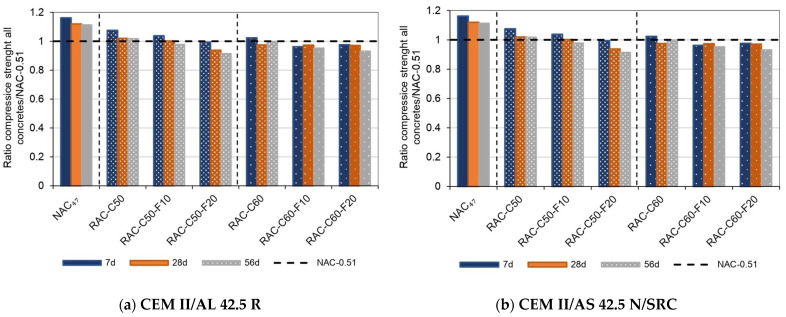

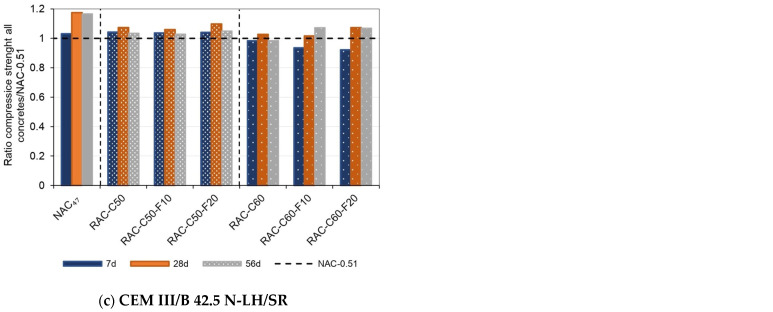
Relative compressive strength at all concrete ages with the following cements: (**a**) Type CEM II/AL; (**b**) Type CEM II/AS; (**c**) Type CEM III/B.

The compressive strength of the RACs was 18% lower than that of NAC-0.47, even though both were produced with the same effective water–cement ratio of 0.47, as shown in Table 5. However, RAC’s compressive strength was comparable to that of NAC-0.51, which had a higher effective water–cement ratio of 0.51 (see Figure 4). These results illustrate the need to adjust the water–cement ratio to attain compressive strength similar to that of NAC-0.51 due to the significant impact of recycled aggregates on concrete’s mechanical properties. As previously demonstrated [51,80], due to the higher porosity and Los Angeles coefficient (weaker particles) of the RCA than those of NA, the RAC produced with 50% CRCA could achieve similar mechanical properties to those of NAC when produced with a lower water–cement ratio. The compressive strength of RACs is influenced by the proportion of CRCA and FRCA employed in concrete production [81,82,83]. For instance, mixes with 50% CRCA (RAC-C50) generally exhibit better performance than those with 60% CRCA (RAC-C60), due to the inferior mechanical quality of the CRCA and potential weaknesses in the old interfacial transition zone (ITZ) or adhered mortar [81,84]. The RAC produced using 10% FRCA achieved acceptable strength, likely owing to the enhanced compaction and improved bonding of the fine fraction with the cement paste [13,85]. This effect was particularly pronounced when RACs were produced using CEM III/B and CEM II/AS cements, which enhance matrix densification and chemical bonding due to their SCMs [86] and the presence of Portland cement mortar in FRCA.

Table 5 demonstrates that the RACs made with CEM II/AL cement obtained a slightly lower compressive strength than those made with CEM II/AS and CEM III/B cements despite having the same water–cement ratio. This difference is attributed to the seasonal production periods; RACs with CEM II/AL were produced in spring/summer, whereas the others were manufactured in autumn/winter, impacting the setting and curing processes of the concrete [87].

Figure 4a–c illustrate the compressive strength ratios of each RAC relative to conventional concrete (NAC-0.51) at 7, 28, and 56 days. In addition, NAC-0.47 achieved a 3% to 17% higher strength than NAC-0.51 at various ages.

Figure 4a (concrete produced using IIAL cement) shows that RAC-C50, RAC-C50-F10, and RAC-C60 achieved similar values to NAC-0.51 at all ages, while RAC-C50-F20 showed a decrease of up to 8.5% at 56 days. Additionally, RAC-C60-F10 and RAC-C60-F20 exhibited reduced early compressive strength (7 days), but this difference was lower at 56 days, achieving a 7% lower strength than NAC-0.51. Figure 4b shows the results for concretes produced with CEM II/AS cement. The concretes produced using 10–20% FRCA obtained a slightly lower strength at 7 days; however, at 28 days, all RACs, including RAC-C60-F20, achieved similar strength to NAC-0.51. At 56 days, the compressive strength of all RACs was between 3% and 7.5% lower. All of the RAC produced using IIIB cement achieved a similar or higher strength than that of NAC-0.51 at 28 and 56 days (see Figure 4c). At 7 days, the concretes produced using 60% CRCA and fine aggregates (10%, 20%) showed lower compressive strength than NAC-0.51.

Furthermore, as discovered in previous studies [13,51], the RAC-C50-F20 concrete (produced with 50% CRCA and 20% FRCA) achieved similar strength to RAC-C50 (using natural sand) and NAC-0.51. However, several researchers [88,89,90,91] have suggested that incorporating up to 30% FRCA as a replacement for natural sand could still achieve satisfactory properties. As mentioned, all of the RACs produced, including RAC-C60-F20, with an effective w/c ratio of 0.47, achieved a similar strength to NAC-0.51 at 28 days. Although the inclusion of FRCA slightly reduced the early strength, which was probably due to the higher water amount caused by the higher WA capacity (old mortar was the main component [92,93]) and weaker bonding of the fine recycled aggregates [30,81,88], this effect was mitigated as the concrete matured, reaching compressive strengths comparable to that of conventional concrete (NAC-0.51) at 56 days. All mixtures achieved a compressive strength of C30/37, which is suitable for structural applications.

#### 3.1.2. Drying Shrinkage

**Figure 5:**
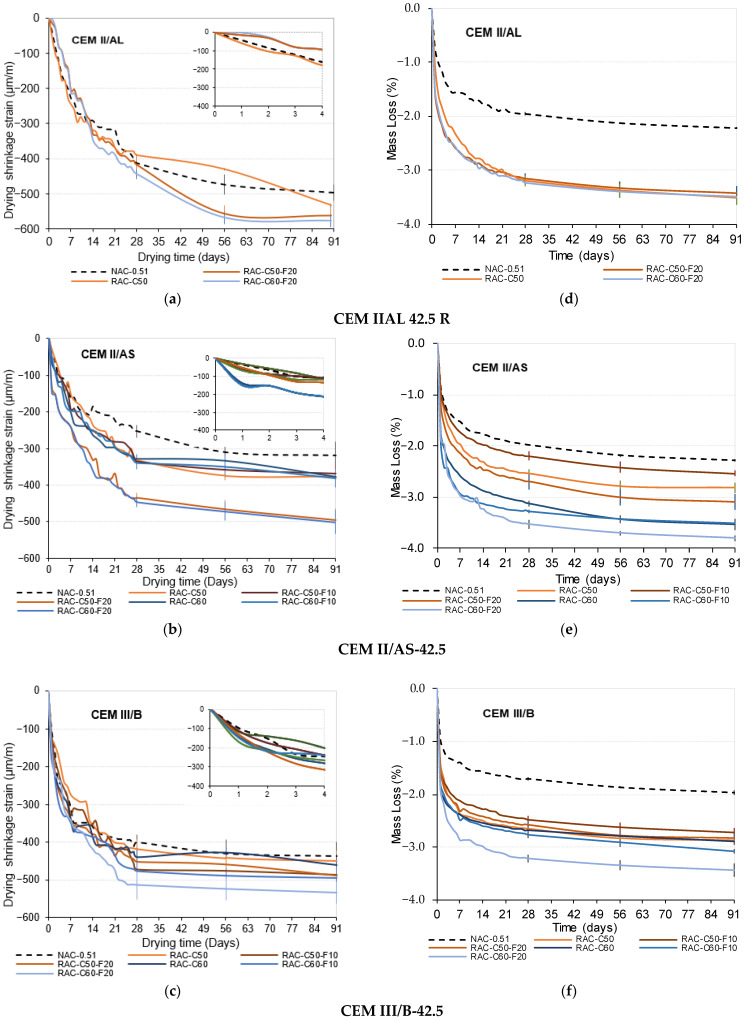
Drying shrinkage value development and mass loss at 91 days and their standard deviation: (**a**,**d**) CEM II/AL, (**b**,**e**) CEM II/AS, (**c**,**f**) CEM III/B.

Figure 5a–f illustrates the values of drying shrinkage (µƐ) and mass loss (%) over 91 days for NAC-0.51 and the RACs produced using CEM II/AL, II/AS, and III/B. In addition, the standard deviation of the shrinkage values are described by the vertical lines.

All concretes produced using CEM II/AL (see Figure 5a) achieved similar shrinkage values (from −496.8 to −576.7 µm/m) at 91 days. The use of FRCA slightly increased the shrinkage value; the RAC-C50-F20 and RAC-C60-F20 concretes exhibited 13.3% and 16.1% higher drying shrinkage, respectively, than that of NAC-0,51, while the RAC-C50 concrete showed a 7.3% higher drying shrinkage than that of NAC-051 (the RAC-C50-F10, RAC-C60, and RAC-C60-F10 concretes were not tested).

The concretes produced using CEM II/AS (Figure 5b) achieved shrinkage values between −318.8 and −501.2 µm/m. The RACs with up to 60% CRCA and 10% FRCA obtained between 15.8% and 19.7% higher drying shrinkage values than NAC-0.51. In addition, the RAC-C50-F20 and RAC-C60-F20 concretes had similar behaviors, with shrinkage values up to 57.2% higher than that of NAC-0.51. However, the values obtained in the RACs were lower than those obtained in concretes produced using IIAL.

All of the concretes produced with CEM III/B (Figure 5c) obtained similar drying shrinkage values ranging from −437.3 to −534 µm/m. RAC-C50 and RAC-C60 achieved 3% and 5% higher shrinkage values, respectively, than NAC-0.51. The use of FRCA increased the shrinkage value of concrete, while RAC-C50-F10, RAC-C50-F20, and RAC-C60-F10 showed 11%, 12%, and 13.4% higher shrinkage values than that of NAC-0.51, and RAC-C60-F20 obtained a 22.1% higher shrinkage value than that of NAC-0.51.

All shrinkage values measured for the RACs were within the acceptable limits according to the guidelines of the American Concrete Institute (ACI) [14], which stipulate an ultimate shrinkage strain of −780 to −800 μm/m, establishing this range as the threshold for drying shrinkage in conventional concrete when a high w/c ratio is used. During the first four days of curing, concretes with lower clinker content (CEM III/B) exhibited the highest shrinkage values (Figure 5c). NAC-0.51 with CEM III/B, CEM II/AL, and CEM II/AS reached −200 µm/m, −170 µm/m, and −110 µm/m, respectively, at 4 days, and it was stabilized over 28 days. Early age drying shrinkage tends to stabilize over time, especially with an SCM as a binder [10,11,51,88]. The shrinkage value increased with higher RCA content, with similar deviations to those of NAC, though with moderate variations in most cases.

Figure 5d–f illustrates the mass loss percentages for the produced concrete mixes. The NAC-0.51 mixes showed mass losses of 2.2% with CEM II/AL, 2.3% with CEM II/AS, and 1.9% with CEM III/B. As expected, the mass loss was greater in mixes with a higher content of RCA (as a consequence of a higher total water amount). The RACs produced using CEM II/AL achieved a mass loss of up to 3.5%, the CEM II/AS mixes obtained values between 2.8% and 3.8%, and the CEM III/B mixes obtained mass losses from 2.86% to 3.4%. These results align with those of prior studies [13,51,67,94,95], confirming that greater drying shrinkage correlates with increased mass loss across similar cement types.

**Figure 6:**
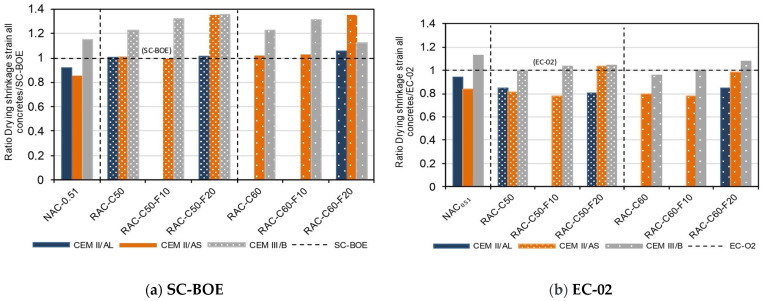
Shrinkage estimation (**a**) experimental results/numerical estimation (SC-BOE); (**b**) experimental results/numerical estimation (EC-02).

In order to validate the drying shrinkage value prediction according to codes, the Structural Concrete Code (SC-BOE) [4] and Eurocode 2: EN 1992-1-1 (EC-02) [6] were used to predict drying shrinkage in RACs, as described in a previous study [13]. SC-BOE [4] does not consider the use of RCA. However, factors such as the compressive strength at 28 days, concrete specimen size, ambient RH, and cement type are considered. CEM IIAL was classified as having a high early strength (Class CR), while CEM II/AS and CEM III/B were classified as having an ordinary early strength (Class CN). Eurocode 2 (EC-02) [6] considers RCA to estimate shrinkage. The influence of CRCA and FRCA was calculated using a factor (ηshRA) in the formula for the drying shrinkage of RAC, defined as 1 + 0.8 αRA, where αRA is the ratio of recycled aggregates (CRCA and FRCA) to total aggregates. This factor is applied when the RCA replaces 20–40% of the NAs (0.20 < αRA ≤ 0.40) [6,13]. In this study, the αRA values were 0.27, 0.31, and 0.36 for the RAC-C50, RAC-C50-F10, and RAC-C50-F20 concretes, respectively, while for the RAC-C60, RAC-C60-F10, and RAC-C60-F20 concretes, the values were 0.32, 0.37 and 0.42. Figure 6 illustrates the ratio between the experimentally obtained drying shrinkage value of each concrete and the value determined using the formulations provided by (a) the Structural Concrete Code (SC-BOE) [4] and (b) Eurocode 2: EN 1992-1-1 (EC-02) [6].

According to Figure 6a, SC-BOE [4] does not accurately estimate the shrinkage value of NAC-0.51 concrete. In order to estimate the shrinkage value, the parameters of the compressive strength at 28 days (as the primary parameter) and the type of cement were considered. However, it was observed that, although the type of cement is considered, SC-BOE does not adequately assess the shrinkage of CEM III/B concrete due to a high early shrinkage caused by the high BFS content in the cement, significantly influencing the total drying shrinkage [10,11]. NAC-0.51 produced with CEM III/B cement had a 15% higher shrinkage value than that estimated using SC-BOE [4]. In addition, NAC-0.51 produced with the CEM II/AL and CEM II/AS cements obtained 9% and 15% lower shrinkage values, respectively, than those calculated using the SC-BOE [4].

SC-BOE accurately estimates the shrinkage of the RAC produced using up to 60% CRCA and 10% FRCA with CEM II/AL and CEM II/AS. However, the RAC-C60-F20 concrete made with CEM II/AL and CEM II/AS achieved 6% and 35% higher shrinkage values, respectively, than the value estimated using the code. In addition, all concrete mixtures produced with CEM III/B achieved higher shrinkage values than those estimated with SC-BOE. Researchers have reported that SC-BOE estimations can have a ±30% dispersion, and they are increased in RACs [9,96]. The higher early shrinkage of concrete produced using a high BFS content influences the total shrinkage [10,11]. This highlights the need to consider other factors for shrinkage estimation, such as environmental conditions, cement types and properties, types of aggregates, and mineral additions [9].

Figure 6b shows the ratio between the experimental results and the EC-02 estimates. EC-02 overestimated the shrinkage values of the NAC-0.51 and RAC concretes produced using CEM II/AL and CEM II/AS. Similarly to SC-BOE, EC-02 underestimated the shrinkage value of NAC-0.51 made with CEM III/B. However, EC-02 accurately predicted shrinkage for the RAC with CEM III/B. EC-02 considers an increase in shrinkage due to recycled aggregates, making its predictions more accurate than those of SC-BOE for drying shrinkage in RACs.

#### 3.1.3. Chloride Ion Penetration

The ASTMC1202 test categorizes chloride ion penetrability into low (1000–2000 Coulombs), moderate (2000–4000 Coulombs), and high (>4000 Coulombs) levels [97]. Table 6 presents the average chloride ion penetrability for the concrete mixtures at 28 and 56 days of curing. This study confirms that chloride ion penetrability significantly varies with the type of cement used, which is consistent with previous findings [57,98,99,100], where a concrete produced with IIAL achieved higher permeability than IIAS concretes and IIIB concretes, which achieved the lowest permeability. In addition, the data in Table 6 reveal that concretes made with CEM II/AL displayed higher variability, as measured with the standard deviation, compared with those with CEM II/AS, while CEM III/B mixtures showed the lowest variability.

Moreover, a direct correlation was found between the RCA replacement ratio and chloride ion penetrability, as reported in earlier studies [31,77,88]. Concrete produced with higher percentages of RCA achieved a higher chloride permeability when the same type of cement was used. However, due to the lower effective water–cement ratio employed in RAC compared with NAC-0.51 (all with similar compressive strength), RAC50 and even the concrete produced with a higher percentage of RCA achieved higher chloride ion penetration resistance than the corresponding NAC-0.51 concrete. Furthermore, the effect of FRCA on chloride penetration was more evident than that of CRCA due to the higher amount of adhered mortar in FRCA [77]. In addition, NAC-0.47, which was produced with a total water–cement ratio of 0.50 as required for the XS1 environment, exhibited the highest chloride ion permeability resistance.

**Figure 7:**
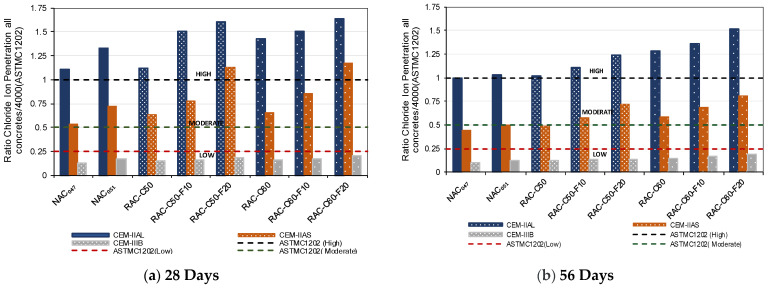
The ratio of chloride ion penetrability (determined in charge passed) for all concretes concerning the maximum value of 4000 Coulombs: (**a**) 28 days and (**b**) 56 days.

Figure 7a,b depicts the ratio between the charge passed by each concrete mix with respect to 4000 Coulombs (the threshold for moderate corrosion risk) at 28 and 56 days, respectively. Figure 7a indicates that all concretes made with CEM II/AL exhibit high chloride ion penetration. The incorporation of BFS into the cement reduced the concrete’s ion penetrability [100]. Although the influence of RCA usage was evident, RAC-C50 (produced with an effective w/c ratio of 0.47) demonstrated lower chloride ion penetrability than NAC (produced with an effective w/c ratio of 0.51), regardless of the cement type used, as reported by Kopecký and Balázs et al. [100]. Furthermore, when FRCA was used, particularly with a 20% replacement of natural sand, the chloride ion penetrability increased, mainly when cement without BFS (CEM II/AL) or with low BFS content (CEM II/AS) was employed. Evangelista and de Brito et al. [31] observed that concrete produced using FRCA exhibited lower resistance to chloride ions than NAC. This is attributed to the high porosity and high volume of adhered mortar in the FRCA, which enhance the permeability to chloride ions [97,101].

Figure 7 also shows that the RAC produced using cement with a greater amount of GGBS achieved the highest chloride ion penetration resistance. Etxeberria et al. [102] reported that concretes mixed with CEM III/B cement and varying recycled aggregate contents (0%, 25%, 50%, 100%) passed charges between 800 and 1400 Coulombs, which was categorized as low chloride ion penetrability. Similarly, Sim and Park [34] noted a minimal impact of FRCA on chloride ion penetration.

At 56 days of curing (Figure 7b), the chloride ion penetration resistance of the produced concretes increased. However, the concretes made using CEM II/AL maintained elevated levels of chloride ion penetrability. This heightened permeability is primarily attributed to the limestone content in the CEM II/AL concrete, which facilitates greater chloride ion migration compared with that in mixes utilizing higher supplementary cementitious material (SCM) substitution [103,104]. The chloride penetrability values observed in this study were marginally lower than those reported by Etxeberria and Castillo [105], where concrete composed of 50% coarse RCA and identical cement—maintaining an effective water–cement ratio of 0.50 and a cement content of 350 kg/m³—demonstrated chloride values of 8799 Coulombs at 28 days and 6377 Coulombs at 56 days. In contrast, Figure 7 shows that the concrete mixes produced using CEM II/AS achieved a decrease in the test value from 12% to 37% from 28 to 56 days, indicating a moderate level of resistance to chloride ion penetration. Additionally, all of the concretes produced with the CEM III/B cement demonstrated consistently low chloride permeability at 28 and 56 days, with a decrease in the test value of 6% to 28% independent of RCA content. Previous findings corroborate that BFS cement notably increases chloride penetration resistance in concrete, primarily through its capacity to immobilize chloride ions [36,37].

#### 3.1.4. Chloride Penetration Depth

**Figure 8:**
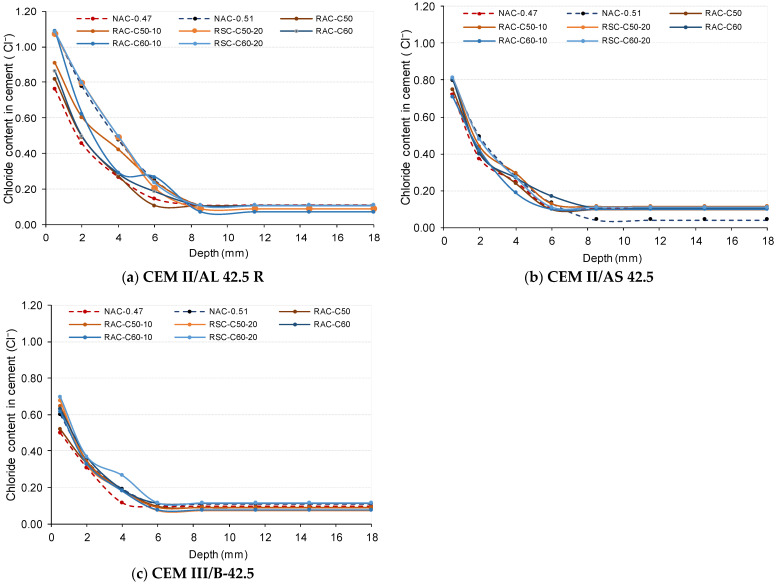
Chloride content per cement weight (%) at different depths of samples: (**a**) CEM II/AL, (**b**) CEM II/AS, (**c**) CEM III/B.

Figure 8 shows the chloride content (as a percentage of cement weight) obtained at different depth layers of samples (depth layers defined in Figure 3) of each concrete mixture after 90 days of exposure to a 3% NaCl solution.

It was observed (Figure 8a) that the concretes produced using IIAL cement achieved the highest chloride content in the first depth layer (L1: 0–1 mm). In addition, the chloride penetrated up to the fifth layer (L5: 7–10 mm deep). At the L5 depth layer, RAC-C50-F20 and RAC-C60-F20, together with NAC-0.51, achieved similar chloride content, which was a higher content than that in RAC-C50-F10 and RAC-C60-F10, but all of these concretes achieved a state of equilibrium at L5. However, the RAC-C50 and NAC-047 concretes had an insignificant or absent chloride amount at the 7–10 mm layer, indicating a state of equilibrium at L4.

In contrast, the concretes produced using CEM II/AS showed intermediate chloride content, in which L4 (5–7 mm deep) was the deepest layer with chloride content. The chloride content at a depth of 6 mm was lower than 0.2%, although the state of equilibrium was achieved at L5. As shown in Figure 8b, the IIAS concretes achieved chloride content at 4 mm depth similar to that obtained at 6 mm in concrete produced with IIAL (Figure 8a). All concretes produced using CEM III/B exhibited very low chloride levels with a chloride content lower than 0.20% at the L3 depth layer (3–5mm), except for RAC-C60-F20. In addition, the absence of chloride—a state of equilibrium—was achieved at 6 mm (L4). The reduced chloride penetration in CEM III/B was due to its high slag content, which provided a denser and less permeable structure, enhancing the binding capacity for chloride ions [29,38].

Fick’s second law controls chloride ion diffusion in concrete, and this is generally considered one-dimensional. Based on the profile data (Figure 8) and following the EN 12390-11 specification, the diffusion coefficient (Dnss) and chloride concentration at the concrete surface $\left(Cs\right)$ were calculated using Fick’s second law in the non-steady state, as shown in Equation (1):

(1) $$C\left(x,t\right)=C0+\left(Cs-C0\right)\times \left[1-erf\left(\frac{x}{2\times \sqrt{Dnss.t}}\right)\right]$$

where: Dnss is the non-steady state diffusion coefficient, expressed in m^2^/s; Cs: is the chloride concentration at the concrete surface； Co: is the initial chloride concentration (chloride content in % mass before immersion in the NaCl solution, which is very low (0.07%) in all cases; $C\left(x,t\right)$ is the chloride content (as a percentage of cement weight, as described in Figure 8) obtained at a given depth x and for a given exposure time; t represents the exposure time in seconds, which in this case, is 7,776,000 s, corresponding to 90 days; x is the depth at which the sample is taken, in millimeters (mm).

In order to carry out the assessment, both the first and last depth layers were not considered, following the EN 12390-11 [106] and SC-BOE [4] standards, which exclude these surface layers from the analysis.

Table 7 describes the Cs (% in the cement mass) and Dnss values obtained in the different concrete mixtures using CEM II/AL, CEM II/AS, and CEM III/B. In all of the concretes produced, the numerically obtained Cs value (see Table 8) was lower than the value described in the first layer (0–1 mm, the point at 0.5 mm depth in Figure 8). Peaking behavior was observed in the 1–3 mm layer in all samples, which was likely due to leaching that caused the decalcification of C-S-H (calcium silicate hydrate), similarly to findings reported in other studies [22].

As shown in Table 7, the highest Cs values were achieved in concretes produced using CEM II/AL, followed by CEM II/AS, and finally, CEM III/B. In addition, the value increased with the amount of RCA used for each corresponding cement type.

Chloride ions can penetrate concrete through diffusion caused by the concentration gradient and capillary suction, which are related to the volume and size of pores and microcracks [107]. The concretes produced using IIAL cement had the highest non-steady state diffusion coefficient (Dnss), ranging from 1.51 × 10^−12^ to 1.97 × 10^−12^ m²/s. The RAC-C50-F20 and RAC-C60-F20 concretes achieved the highest surface chloride concentrations and diffusion coefficients, indicating the lowest resistance to chloride penetration.

On the other hand, the Dnss coefficients of the CEM II/AS concretes ranged from 1.1 × 10^−12^ to 1.31 × 10^−12^ m²/s, while the lowest values were observed in CEM III/B at around 7 × 10^−13^ m²/s. In addition, the RAC produced using IIIB cement obtained a lower Dnss value than the NAC-0.51 concretes produced using IIAS and IIAL. All of the concretes produced with CEM III/B had values that fell within the ranges reported by other authors in the literature [108,109]. Chloride can accumulate in the diffuse layer of the C-A-S-H in the concretes produced using SCMs. This accumulation is usually referred to as physical binding [38]. In addition, concretes with SCMs have a lower portlandite (CH) content due to the reduced clinker content, which enhances their resistance to leaching and chloride penetration.

The values obtained in this study are consistent with typical values reported according to the NT Build 443 test method, as detailed in [108,109] and the work of Nilson et al. [110]. Maes et al. [109] studied mixtures with a w/c ratio of 0.45 in which it was observed that at 84 days, the non-steady state diffusion coefficients (Dnss) ranged from 3.72 × 10^−12^ to 5.27 × 10^−12^ m²/s for Portland cement (PC), from 5.11 × 10^−12^ to 5.97 × 10^−12^ m²/s for sulfate-resistant cement (HSR), and from 2.33 × 10^−12^ to 2.94 × 10^−12^ m²/s for mixtures with 50% blast-furnace slag (S50). Moreover, in a study by Moreno et al.[111], for mixtures with a w/c ratio of 0.5, the inclusion of construction and demolition waste (CDW), which was used as a partial replacement for cement, improved the chloride resistance. The diffusion coefficients were 2.9 × 10^−11^ m²/s (calcareous fines-glass, 7% Hc-G) and 1.5 × 10^−11^ m²/s (siliceous fines-glass, 7% Hs-G), in comparison with the reference PC cement, which had a diffusion coefficient of 4.3 × 10^−11^ m²/s. In a study carried out in bridges [110], the chloride diffusion coefficients ranged from 0.02 to 0.8 × 10^−12^ m²/s, indicating variations in resistance to chloride penetration depending on the age and type of concrete. These values reflect differences that are attributable to environmental exposure and the specific characteristics of each concrete mix.

**Figure 9:**
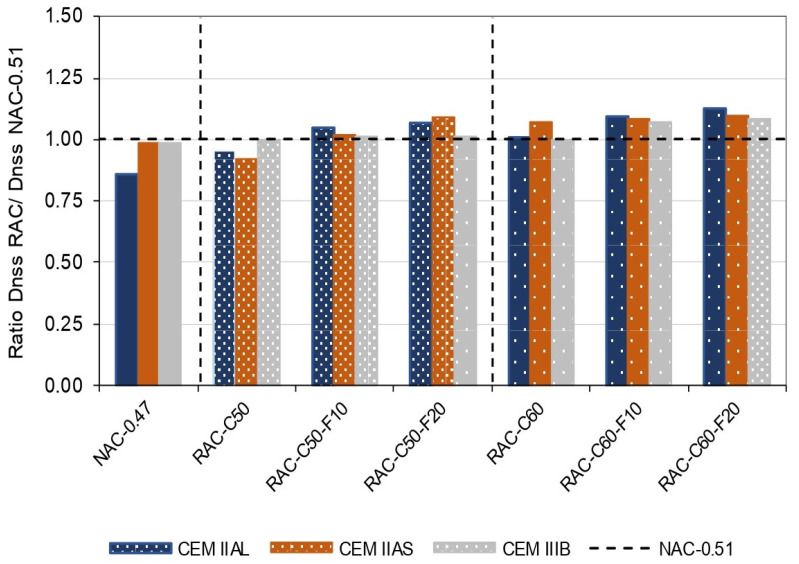
Ratio of the non-steady-state diffusion coefficients (Dnss) of RACs with respect to NAC-0.51.

Figure 9 shows the influence of RCA on the Dnss value of RAC with respect to that of NAC-0.51 when they were produced using different cements. It shows that concrete mixes with 50% CRCA achieved a higher chloride penetration resistance than that of NAC-0.51 concrete produced using the three types of cements. According to SC, in the XS1 environment, the maximum total water–cement ratio must be 0.50 for NAC, corresponding to an effective 0.47, which is the effective water–cement ratio used in RAC. In addition, it is observed that while NAC-0.47 produced using II/AL cement obtained lower chloride penetration than the NAC-0.51 concrete, the NAC-0.47 and NAC-0.51 concretes produced with IIAS and IIIB cements achieved similar chloride penetration.

Moreover, the mixtures containing 60% CRCA and 20% FRCA demonstrated that the type of cement has a significant impact on enhancing durability under adverse conditions. In particular, mixtures incorporating CEM III/B cement (Table 7), with high additions of slag, have proven to be especially effective in reducing the chloride diffusion coefficient.

### 3.2. Carbonation Resistance

The natural and accelerated carbonation rates were determined in the concrete mixtures produced using specific carbonation processes. In addition, accelerated carbonation processes were used to estimate the theoretical natural carbonation rates of the different concretes, and they were then compared with the natural carbonation rate obtained in the natural process.

#### 3.2.1. Accelerated Carbonation

Table 8 describes the average carbonation depth after 91 days for NAC-0.51 and RAC (which obtained similar compressive strength), as well as NAC-0.47 and it shows their accelerated carbonation coefficient (Kacc) according to the EN 12390-12 specifications.

The Kacc value of each concrete was calculated under steady-state condition based on Fick’s first law of diffusion, represented by Equation (2).

(2) $$Xc(t)=Kacc\cdot {\left(t\right)}^{0.5}$$

Here, Xc is the determined carbonation depth (mm), Kacc is the accelerated carbonation coefficient (mm/day^0.5^), and t is time (days). The carbonation depth was determined at 0, 14, 28, 56, 70, and 91 days of exposure to 3% CO_2_, 57% RH, and 20 °C.

The NAC-0.47 concrete had the lowest carbonation depths in each cement and concrete type. Considering the concretes with the same compressive strength, NAC-0.51, RAC-C50 and RAC-C60 achieved similar carbonation depth in all the cement and concrete types. As mentioned for compressive strength, by lowering the water-to-cement (w/c) ratio, RAC achieved carbonation depths comparable to those of NAC [51,77]. However, the use of 20% FRCA in concrete production increased the carbonation depth.

The concretes produced with the CEM II/AS cement exhibited the lowest Kacc values, followed by those made with the CEM II/AL and CEM III/B cements. These findings are consistent with previous results [51,112], which demonstrated that concretes incorporating CEM II/AS consistently display the lowest carbonation depths, regardless of the aggregates used. In addition, all of the RACs produced with IIAS and IIAL cements achieved lower Kacc values (accelerated carbonation rate) than NAC-0.51 produced with IIIB cement. Further investigations [46,53] revealed that the increased carbonation coefficient observed in concretes with the CEM III/B cement was intrinsically linked to its lower clinker content relative to the CEM II types. This characteristic reduces CO_2_ buffering capacities, primarily due to the reduced calcium oxide (CaO) content. As such, replacing cement with a substantial volume of mineral admixtures, which inherently lowers the CaO content, correspondingly decreases the carbonation resistance of the recycled concrete [34,46,48]. In addition, the concretes produced with CEM II/AS achieved a lower carbonation rate than those produced with CEM II/AL due to the higher carbonate CaO availability in CEM II/AS.

**Figure 10:**
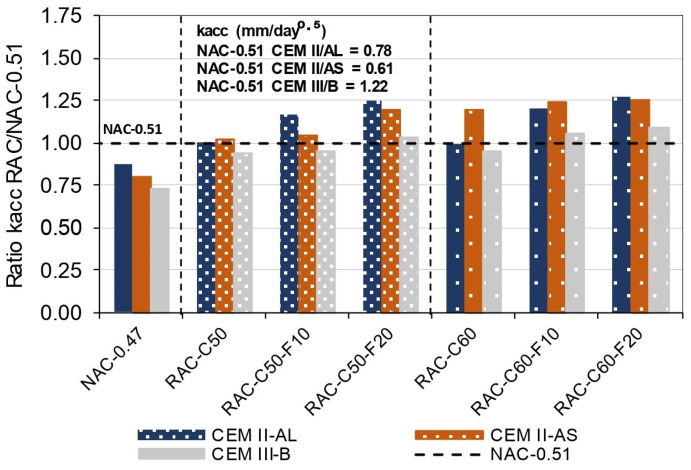
Ratio of the RAC Kacc to the NAC-0.51 Kacc.

The results shown in Figure 10 illustrate the relationship between the Kacc values of RAC and NAC 0.51 in each type of cement concrete. This relationship demonstrates that the RAC produced using CEM II/AS and II/AL achieved a comparable performance and higher carbonation rate than that of NAC-0.51. In contrast, the RAC produced with CEM III/B achieved similar values to those of NAC-0.51.

The RAC-C50 concretes achieved less than a 3% increase in Kacc compared with NAC-0.51 in every cement type. The RAC-C60 concretes also showed a similar or slightly superior carbonation rate to that of NAC-0.51, except for the concrete produced with IIAS, which achieved 20% higher Kacc than that of NAC-0.51. These trends are consistent with findings from prior research [44,45,46,105]. In addition, the use of 10% FRCA with 50% CRCA in RAC-C50-F10 resulted in a slight 5% increase in Kacc, except for the one produced with CEM II/AL, which achieved a 17% increase, compared with the NAC-0.51 concrete.

In contrast, the RAC-C60-10 concretes produced with the CEM II/AL, CEM II/AS, and CEM III/B cements achieved 21%, 24%, and 5% higher Kacc values than that of the corresponding NAC-0.51. Moreover, the use of 20% FRCA in the substitution of natural sand led to substantial increases in Kacc in comparison with the values obtained for NAC, especially in concretes produced with IIAL and IIAS cement, which achieved up to 25% higher Kacc values than those of NAC-0.51 concretes. The lower performance of RAC than that of NAC can be attributed to the higher porosity of recycled aggregates, a factor that was especially pronounced in the case of FRCA [88].

The Kacc value can be employed to estimate the theoretical natural carbonation coefficient (knatTHEO) (Table 8) [113,114] of each type of concrete produced using Equation (3).

(3) $$\frac{Kacc}{KnatTHEO}=\frac{{\left(\emptyset acc\right)}^{0.5}}{{\left(\emptyset natTHEO\right)}^{0.5}},$$

Here, Kacc and knatTHEO are the CO_2_ concentrations in the accelerated carbonation (3%) and natural carbonation processes (430 ppm, in Barcelona), respectively.

The obtained values of KnatTHEO were compared with the natural carbonation rate that was determined experimentally.

#### 3.2.2. Natural Carbonation Resistance

**Figure 11:**
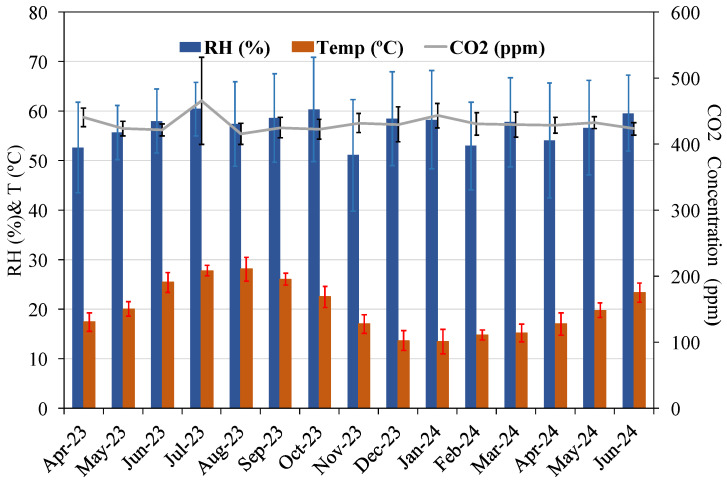
Environmental conditions for natural carbonation.

After four days of curing, the produced concrete specimens were exposed for one year under natural environmental conditions in Barcelona with an average of 431 ppm CO_2_, 56.8% RH, and 20.1 °C, as illustrated in Figure 11. The average carbonation depth data after one year of exposure and the calculated natural carbonation rate (knat) for the NAC and RAC concretes are described in Table 9.

The NAC-0.51 concretes achieved a similar or slightly higher carbonation depth than that of NAC-0.47 and RAC in each corresponding cement type. The influence of the cement type was apparent; concretes made with the CEM III/B cement showed significantly higher carbonation depth, followed by those with CEM II/AL. Moreover, concretes with CEM II/AS achieved the lowest carbonation depth, which was similar to the accelerated carbonation process. In concrete produced using the IIIB cement, with a large amount of SCMs, due to the reduction of portlandite (available CaO) and the lower pH buffering capacity, the susceptibility to carbonation was increased [40]. While CEM II/AS and CEM II/AL have similar clinker amounts (approximately 88%), IIAS has more hydraulic CaO available, which gives it a higher carbonation resistance than IIAL. The IIAL contains 10% limestone filler [40,115,116,117].

**Figure 12:**
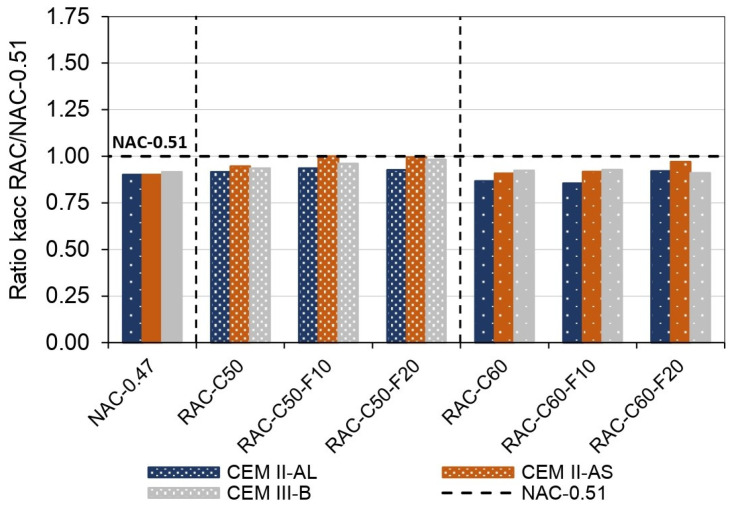
Ratio of the RAC Knat to the NAC-0.51 Knat.

Figure 12 describes the ratio between the Knat coefficient of RAC with respect to that of NAC-0.51 (concretes with similar compressive strength) for each corresponding cement type. It shows that the RAC achieved a lower Knat value than that of NAC-0.51. This behavior differed from the accelerated carbonation process in which the Kacc value of RAC was higher than that of NAC-0.51. The concrete specimens were exposed to natural carbonation after 4 days of curing, which was probably due to water inside the RCA, which helped with internal curing and reduced the carbonation rate [118,119,120]. In addition, the higher calcium availability in RAC could influence the improvement of the carbonation resistance of RAC with respect to NAC-0.51.

#### 3.2.3. Knat vs. KnatTHEO

Table 10 shows the ratio between KnatTHEO (see Table 8) and Knat (see Table 9) of the concretes obtained through accelerated and natural carbonation processes, respectively. The results indicate that the Knat coefficient was between 2.0 and 2.8 times higher in NAC and between 1.6 and 2.4 times higher in RAC compared with the KnatTHEO coefficient value. These findings align with those of other studies [112], which report that the experimentally determined natural carbonation coefficient (Knat) is 1.6 to 1.8 times higher than the carbonation rate estimated using an accelerated test. The concretes were exposed to natural carbonation between May and June (at 20–26 °C and 55% RH) after only 4 days of curing, which increased the carbonation rate of the concretes and, more importantly, of NAC (RAC may achieve internal curing). Moreover, Knat was determined using only data from 1 year of exposure, and it may be reduced after a longer period of exposure [121].

Tests have demonstrated that the carbonation depths in both RAC and NAC increase when samples are cured shortly in a humid room and undergo a long curing process in drier environments, highlighting the importance of curing conditions [20,122]. The natural carbonation, influenced by both the duration of exposure and interaction with realistic environmental cycles, results in deeper penetration of CO_2_ into concrete compared with that in the controlled environments of accelerated tests [20,123].

**Figure 13:**
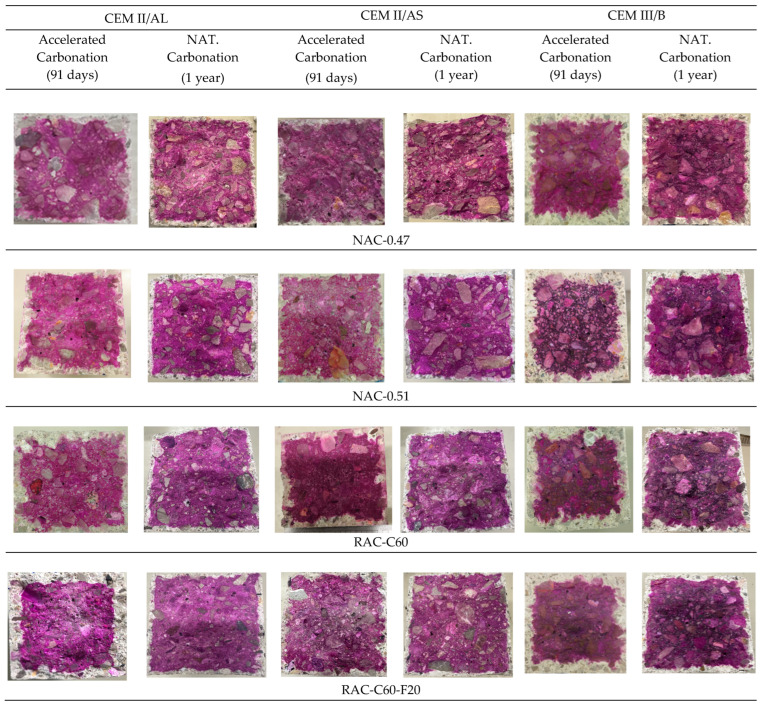
Visual comparison of the carbonation depth in natural (1 year) and accelerated conditions (91 days) for concretes with different cement types.

Figure 13 visually compares the carbonation depth obtained in the NAC-0.47, NAC-0.51, RAC-C60 and RAC-C60-F20 (with RAC having the highest percentages) concretes produced using different types of cement under natural conditions over one year versus under accelerated conditions over 91 days.

#### 3.2.4. Carbonation Analysis at 50 and 100 Years

Table 11 details the predicted carbonation depth for each type of concrete calculated using the Knat value over 50 and 100 years of expected service lives. The Spanish Structural Concrete Code (SC-BOE) [4] specifies the minimum cover depth for concrete structures exposed to XC3 and XC4 environments, corresponding to a design life of 50 and 100 years. For XC3 exposure conditions, a minimum cover of 20 mm and 30 mm is required for a 50 and 100 year lifespan, respectively. For XC4 exposure conditions, the required minimum cover increases to 25 mm and 35 mm, respectively. In addition, EC-02, as shown in Table 11 [6], describes that the minimum cover—given the data from SC-BOE [4]—should be increased by +5 mm when recycled aggregates are used.

**Figure 14:**
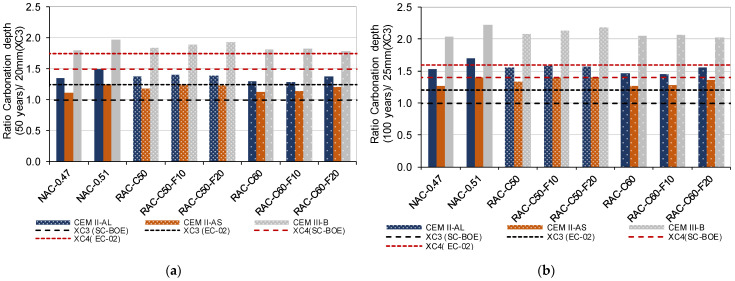
Comparison of the carbonation depth relative to exposure class XC3 for all concretes (**a**) 50 years, (**b**) 100 years.

According to the results (Table 11 and Figure 14), all of the concretes produced using CEM II/AL and CEM II/AS achieved 50 years of service life with a minimum cover of 30 mm defined for an XC4 environment (SC-BOE [4] regulations). Although a minimum cover of 20 mm was generally insufficient for XC3 conditions, CEM II/AS adequately met the 50-year service life requirements according to the EC-02 recommendation. In addition, in the concretes produced with CEM II/AS and CEM II/AL, the minimum cover of 35 + 5 mm was adequate for 100 years of service life in an XC4 environment (SC-BOE [4] and EC-02 [6] regulations). In all of the concretes mentioned, the RAC achieved similar or even higher carbonation resistance than the NAC.

Figure 14 shows the ratio of carbonation depths after a lifespan of 50 and 100 years (values described in Table 11) with respect to the minimum cover defined by SC-BOE for XC3 (20 mm and 25 mm). In addition, the ratios of the minimum cover requirements of XC3 (EC-02), XC4 (SC-BOE) and X4 (EC-02) with respect to that of XC3 (SC-BOE) are described.

## 4. Conclusions

The following conclusions can be drawn from the results of this study:

- This study demonstrates that incorporating up to 60% CRCA and 20% FRCA can result in compressive strength levels comparable to those of NAC by lowering the RAC’s water–cement ratio by approximately 0.04. This underscores the potential of RCA as a sustainable alternative while maintaining structural integrity.
- Regarding the drying shrinkage:
  ○ All RAC produced, with a maximum of 60% CRCA and 20% FRCA and independently of the cement type employed (IIAL, IIAS and IIIB), achieved admissible values at 91 days (maximum of −580 μm/m);
  ○ The concrete produced using up to 60% CRCA achieved a similar shrinkage value to that of NAC-0.51 concrete produced with the same cement. However, the concrete using 20% FRCA (with 60% of CRCA) made with CEM II/AL, CEM II/AS, and CEM IIIB achieved 16.1%, 57.2%, and 22.1%, respectively, higher shrinkage than that of the NAC. However, all achieved lower drying shrinkage values than the acceptable values defined by the ACI (up to −800 μm/m);
  ○ EC-02 is more accurate than SC-BOE in predicting the drying shrinkage of RAC regardless of the cement type used. Both standards fail to accurately estimate NAC-0.51 produced with CEM III/B, as they prioritize 28-day compressive strength over the initial shrinkage values. This underscores the need for more comprehensive models incorporating environmental factors and different cement and aggregate types.
- Regarding the durability properties:
  ○ RACs with 50% and 60% CRCA (produced with an effective w/c ratio of 0.47) exhibit similar or higher resistance to chloride ion penetration and carbonation in comparison with NAC-0.51 (effective w/c ratio of 0.51) when they have similar compressive strengths;
  ○ The concretes (including the RACs) produced with CEM III/B obtained a low chloride concentration at the concrete surface (Cs) and low non-steady state diffusion coefficient (Dnss) values. However, the use of 20% FRCA increased chloride ion penetration;
  ○ RAC-C60-F20 achieves high durability in chloride-aggressive environments when it is produced using cements with high BFS content, such as CEM III/B. In addition, it achieves higher chloride resistance than that of NAC produced using CEM II/AL and CEM II/AS;
  ○ RAC-C60-F10 produced with CEM II/AS achieves moderate chloride ion penetrability resistance, while any concrete, including NAC, produced with IIAL presents no resistance to chloride ions;
  ○ The concretes made with CEM II/AS, followed by CEM II/AL and CEM III/B, achieved the highest carbonation resistance, independent of the type of aggregates used;
  ○ The use of 20% FRCA increased the carbonation rate (up to 25%) compared with NAC-0.51 when an accelerated carbonation test was carried out. However, under natural conditions, these RAC concretes exhibited a lower carbonation rate than that of NAC-0.51;
  ○ The obtained natural carbonation rate (Knat) values were between 2.0 and 2.8 times higher for NAC and between 1.6 and 2.4 times higher for RAC than the theoretical natural carbonation rate (knatTHEO) obtained from the accelerated carbonation test.

Recycled concrete with up to 60% CRCA and 20% FRCA achieved an adequate drying shrinkage value and showed satisfactory durability performance in XC1 to XC4 and XS1 environments, depending on the type of cement used:

○ RAC-C60-F20 produced with CEM II/AS cement achieved adequate carbonation resistance in XC3 and XC4 environments, ensuring a service life of 50 years. However, for chloride resistance, only concrete with up to 60% CRCA and 10% FRCA exhibited moderate chloride ion penetration values and lower chloride diffusion coefficients (Dnss) than those of CEM II/A-L cement;
○ RAC-C60-F20 produced with CEM II/AL cement, while providing adequate carbonation resistance in XC4 environments, presented high chloride ion penetration and a high Dnss value, with values slightly higher than those of NAC-0.51;
○ RAC-C60-F20 produced with CEM III/B exhibited low carbonation resistance and very high chloride penetration resistance, as indicated by lower surface chloride concentrations (Cs) and reduced Dnss values, similarly to NAC-0.51.

For future research, it is recommended that the long-term durability and resistance to environmental factors such as corrosion and carbonation in structural concretes made using RCA are explored. This investigation should focus on RAC with a similar compressive strength to that of NAC, which can be achieved by adjusting the effective water–cement ratio in RAC mixes. Furthermore, it is crucial to delve deeper into the analysis of concrete produced using 60% CRCA and 20% FRCA, evaluating its behavior under various environmental and exposure conditions, including real-scale tests.

## Figures and Tables

**Table 1:** Composition of cement as a percentage of the total weight.

Cement	CaO	SiO2	Al2O3	Fe2O3	SO3	MgO	K2O	TIO2	Na2O
CEM II/AL 42.5 R	61.47	17.87	3.61	2.64	3.69	1.45	0.736	0.183	0.228
(88% clinker, 12% limestone, excluding the set regulator, added in 5%)									
High initial strength, ideal for applications requiring rapid setting									
CEM II/AS 42.5 N/SRC	59.97	21.66	4.35	3.75	3.47	2.1	0.395	0.327	0.314
(83% clinker, 12% blast furnace slag (BFS) and 5% minority component)									
Providing moderate sulfate resistance and enhanced durability									
CEM III/B 42.5 N-LH/SR	49.4	27.8	8.41	1.96	3.96	4.65	0.48	0.457	0.365
(27% clinker, 70% BFS and 3% minority component)									
Low heat development and sulphate resistance									

**Table 2:** Properties of the natural and Type A RCAs studied.

Property	Density (Kg/m^3^)	Water Absorption (%)	Humidity (%)	Sand Equivalent (%)	Los Angeles Coefficient (%)	Flakiness Index (%)	Alkali–Aggregate Reaction (%)
Standard	EN 1097-6 [62]	EN 1097-6 [62]		EN-933-8 [63]	EN 1097-2 [64]	EN 933-3 [65]	UNE-146508 [66]
FNA (0/4)	2.67	0.95	0.37	100			
CNA-1 (4/10)	2.65	0.77	0.16				
CNA-2 (8/20)	2.68	0.73	0.1				
FRCA (0/4)	2.32	5.73	2.73		35.77		0.042
CRCA-1 (2/10)	2.33	5.62	4.50				
CRCA-2 (8/20)	2.36	5.16	4.55			12.81	
SC-BOE ^1^	2.1 *	<7		>70	<40	<35	<0.10

^1^ Requirements defined in Structural Concrete Code (SC-BOE). * Property defined in EN 206.

**Table 3:** Mix proportions for 1 m^3^ concretes produced with CEM II/AL, CEM II/AS and CEM III/B.

Materials	Concrete Types							
(kg)	NAC-0.51	NAC-0.47	RAC-C50	RAC-C50-F10	RAC-C50-F20	RCA-C60	RCA-C60-F10	RCA-C60-F20
Cement	300	300	300	300	300	300	300	300
Total water	165	150	175.8	179.5	182.3	180.8	184.34	187.48
CNA-1	354.5	360.1	180.5	180.5	180.5	144.4	144.4	144.4
CNA-2	723.68	737.2	369.3	369.3	369.3	295.5	295.5	295.5
FNA	954.1	971.9	1014.2	875.7	778.4	973	875.7	778.4
CRCA-1	-	-	165.8	165.2	165.6	198.9	198.6	199.7
CRCA-2	-	-	338.8	339.1	337.2	406.0	406.1	404.2
FRCA	-	-	-	87.1	174.1	-	87.1	174.1
P (%)	1/0.7 ^1^	1	1/0.6 ^1^	1/0.5 ^1^	1/0.3 ^1^	1/0.3 ^1^	1/0.3 ^1^	1/0.3 ^1^
S (%)	1	1	1/1.5 ^1^	1/1.5 ^1^	1/1.5 ^1^	1/1.5 ^1^	1/1.5 ^1^	1/1.5 ^1^
effective w/c	0.51	0.47	0.47	0.47	0.47	0.47	0.47	0.47
Slump-IIAS (mm)	175	145	150	155	150	175	155	160
Slump-IIIB (mm)	175	160	135	150	150	140	150	125
Slump-IIAL (mm)	175	150	190	200	195	180	210	195

^1^ Plasticizer content utilized in CEM II/AL.

**Table 5:** Compressive strength and its standard deviation (values in brackets) in all of the produced concrete.

Concrete Reference	IIAL			IIAS			IIIB		
	7d	28d	56d	7d	28d	56d	7d	28d	56d
NAC-0.47	52.5 (1.3)	62.9 (1.3)	65.5 (1.0)	54.5 (1.3)	69.8 (1)	71.3 (1.9)	53.1 (0.8)	67.2 (0.4)	69.9 (0.9)
NAC-0.51	45.2 (2.0)	56.2 (1.6)	58.8 (1.0)	54.1 (2.0)	59.2 (0.5)	64.7 (0.2)	51.5 (0.1)	57.2 (1.2)	59.9 (0.9)
RAC-C50	48.6 (2.5)	57.3 (1.0)	59.9 (1.7)	53.9 (2.5)	59.2 (2.3)	62.8 (1.2)	53.7 (0.3)	61.4 (3.0)	62.0 (1.0)
RAC-C50-F10	46.9 (1.8)	56.3 (1.5)	57.5 (0.3)	52.4 (1.8)	59.7 (1.3)	59.9 (0.2)	53.4 (2.8)	60.6 (1.7)	61.6 (0.7)
RAC-C50-F20	44.9 (2.4)	52.7 (0.3)	53.8 (0.4)	50.2 (2.4)	60.7 (0)	63.7 (1.2)	53.6 (1.5)	62.8 (1.4)	62.9 (1.3)
RAC-C60	46.3 (0.1)	54.8 (0.8)	58.8 (0.3)	51.6 (1.2)	60.2 (0.9)	60.9 (0.2)	50.7 (0.9)	58.8 (0.6)	59 (0.1)
RAC-C60-F10	43.5 (0.6)	54.7 (1.0)	56.1 (0.0)	49.8 (0.5)	59.2 (0.4)	62.2 (0.3)	48.2 (1.6)	58.2 (2.0)	64.3 (1.9)
RAC-C60-F20	44.1 (0.6)	54.6 (0.8)	54.8 (0.1)	45.1 (0.9)	58.7 (0.8)	61.7 (2.0)	47.5 (1.2)	61.4 (0.5)	64.1 (0.9)

Compressive strength values for 100 mm cubic specimens. () Standard deviation.

**Table 6:** Chloride ion penetrability and standard deviation as determined in charge passed in coulombs.

	IIAL			IIAS			IIIB		
Concrete Types	(Coulombs)		∆ (%)	(Coulombs)		∆ (%)	(Coulombs)		∆ (%)
	28d	56d		28d	56d		28d	56d	
NAC-0.47	4451 (194)	3971 (94)	11	2145 (281)	1766 (44)	18	530 (2)	408 (5)	23
NAC-0.51	5314 (2)	4096 (271)	23	2897 (111)	1976 (129)	32	674 (15)	501 (12)	26
RAC-C50	4479 (441)	4065 (71)	9	2535 (136)	1962 (80)	23	610 (9)	503 (8)	18
RAC-C50-F10	6038 (596)	4448 (97)	26	3130 (58)	2293 (5)	27	626 (16)	531 (15)	15
RAC-C50-F20	6401 (569)	4944 (178)	23	4515 (91)	2866 (66)	37	740 (40)	532 (18)	28
RAC-C60	5726 (250)	5140 (636)	10	2648 (71)	2329 (28)	12	651 (4)	570 (4)	12
RAC-C60-F10	6009 (197)	5454 (33)	9	3425 (78)	2736 (64)	20	707 (44)	668 (14)	6
RAC-C60-F20	6549 (567)	6048 (43)	8	4696 (17)	3239 (119)	31	843 (69)	767 (3)	9

Standard deviation (values are given in brackets).

**Table 7:** Chloride concentration at the concrete surface (Cs) and non-steady state diffusion coefficient (Dnss).

	CEM II/AL			CEM II/AS			CEM III/B		
	Cs	Dnss		Cs	Dnss		Cs	Dnss	
	%	m^2^/s	R^2^	%	m^2^/s	R^2^	%	m^2^/s	R^2^
NAC-0.47	0.483	1.51 × 10^−12^	0.95	0.49	1.18 × 10^−12^	0.95	0.35	7.00 × 10^−13^	0.89
NAC-0.51	0.748	1.75 × 10^−12^	0.98	0.61	1.19 × 10^−12^	0.99	0.46	7.10 × 10^−13^	0.98
RAC-C50	0.697	1.68 × 10^−12^	0.89	0.58	1.10 × 10^−12^	0.95	0.47	7.07 × 10^−13^	0.99
RAC-C50-F10	0.765	1.83 × 10^−12^	0.97	0.58	1.22 × 10^−12^	0.96	0.48	7.19 × 10^−13^	0.98
RAC-C50-F20	0.977	1.87 × 10^−12^	0.95	0.64	1.30 × 10^−12^	0.93	0.49	7.18 × 10^−13^	0.98
RAC-C60	0.751	1.76 × 10^−12^	0.90	0.56	1.28 × 10^−12^	0.99	0.50	7.09 × 10^−13^	0.98
RAC-C60-F10	0.768	1.91 × 10^−12^	0.94	0.60	1.29 × 10^−12^	0.88	0.52	7.61 × 10^−13^	0.98
RAC-C60-F20	0.984	1.97 × 10^−12^	0.95	0.66	1.31 × 10^−12^	0.84	0.54	7.68 × 10^−13^	0.96

**Table 8:** Carbonation depth and accelerated carbonation coefficient of all concrete (standard deviation values are given in brackets).

Concrete Types	Carbonation Depth(mm) at 91 Days			Carbonation Coefficient					
				Kacc (mm/day^0.5^)			KnatTHEO (mm/year^0.5^)		
	II AL	II AS	III B	II AL	II AS	III B	II AL	II AS	III B
NAC-0.47	6.5 (0.1)	4.9 (0.2)	10 (0.3)	0.68	0.49	0.90	1.55	1.12	2.06
NAC-0.51	7.7 (0.1)	6 (0.1)	12 (0.4)	0.78	0.61	1.22	1.78	1.39	2.80
RAC-C50	8 (0.4)	6.1 (0.2)	12.1 (0.2)	0.78	0.62	1.15	1.78	1.43	2.62
RAC-C50-F10	8.7 (0.2)	6.3 (0)	12.1 (0.1)	0.91	0.64	1.16	2.09	1.46	2.64
RAC-C50-F20	9.8 (0)	7.2 (0.2)	12.9 (0.1)	0.97	0.73	1.26	2.22	1.66	2.87
RAC-C60	7.3 (0.5)	6.34 (0.3)	11.8 (0.2)	0.77	0.65	1.17	1.76	1.49	2.67
RAC-C60-F10	9.3 (0)	7.41 (0.1)	12.18 (0.1)	0.94	0.75	1.29	2.15	1.72	2.95
RAC-C60-F20	10.4 (1.6)	7.53 (0)	12.59 (0.1)	0.99	0.77	1.34	2.28	1.75	3.06

**Table 9:** Carbonation depths and natural carbonation coefficient of all concretes (standard deviation values are given in brackets).

Concrete Types	Carbonation Depth (mm) at 1 Year			Carbonation Coefficient		
				Knat (mm/year^0.5^)		
	II AL	II AS	III B	II AL	II AS	III B
NAC-0.47	4.2 (0)	3.5 (0.04)	5.1 (0.09)	3.82	3.16	5.08
NAC-0.51	4.2 (0.02)	3.7 (0.04)	5.7 (0.11)	4.24	3.50	5.55
RAC-C50	4.2 (0.02)	3.5 (0.42)	5.2 (0.15)	3.89	3.32	5.19
RAC-C50-F10	4.2 (0.15)	3.7 (0.04)	5.3 (0.1)	3.97	3.50	5.33
RAC-C50-F20	4.2 (0.09)	3.6 (0.09)	5.4 (0.31)	3.93	3.49	5.45
RAC-C60	4.0 (0.11)	3.5 (0.04)	5.1 (0.04)	3.68	3.18	5.12
RAC-C60-F10	4.1 (0.02)	3.5 (0.02)	5.1 (0.04)	3.63	3.21	5.15
RAC-C60-F20	4.2 (0.15)	3.7 (0.15)	5.2 (0.04)	3.90	3.40	5.05

**Table 10:** Relationship between Natural Carbonation and Accelerated Carbonation in Concretes with NA and RCA.

Knat and KnatTHEO Relationship			
MIX	CEM II/AL	CEM II/AS	CEM III/B
NAC-0.47	2.5	2.8	2.5
NAC-0.51	2.4	2.5	2.0
RAC-C50	2.2	2.3	2.0
RAC-C50-F10	1.9	2.4	2.0
RAC-C50-F20	1.8	2.1	1.9
RAC-C60	2.1	1.9	1.9
RAC-C60-F10	1.7	1.9	1.7
RAC-C60-F20	1.7	1.9	1.6

**Table 11:** Carbonation depth after a lifespan of 50 and 100 years.

Concrete Types		k_nat_ (50 years)			k_nat_ (100 years)		
		II AL	II AS	III B	II AL	II AS	III B
NAC-0.47		27.0	22.3	35.9	38.2	31.6	50.8
NAC-0.51		30.0	24.8	39.2	42.4	35.0	55.5
RAC-C50		27.5	23.5	36.7	38.9	33.2	51.9
RAC-C50-F10		28.1	24.8	37.7	39.7	35.0	53.3
RAC-C50-F20		27.8	24.6	38.5	39.3	34.9	54.5
RAC-C60		26.0	22.5	36.2	36.8	31.8	51.2
RAC-C60-F10		25.6	22.7	36.4	36.3	32.1	51.5
RAC-C60-F20		27.6	24.0	35.7	39.0	34.0	50.5
Min. Cover (mm)	XC3		20 + 5 *		25 + 5 *		
	XC4		30 + 5 *		35 + 5 *		

* EC-0.2 recommends increasing the minimum cover by 5 mm when recycled aggregates are used.

## Data Availability

Data are contained within the article.
